# Traditional Processed Meat Products Re-designed Towards Inulin-rich Functional Foods Reduce Polyps in Two Colorectal Cancer Animal Models

**DOI:** 10.1038/s41598-019-51437-w

**Published:** 2019-10-15

**Authors:** Javier Fernández, Estefanía Ledesma, Joaquín Monte, Enric Millán, Pedro Costa, Vanessa García de la Fuente, María Teresa Fernández García, Pablo Martínez-Camblor, Claudio J. Villar, Felipe Lombó

**Affiliations:** 1 Research Unit “Biotechnology in Nutraceuticals and Bioactive Compounds-BIONUC”, Departamento de Biología Funcional, Área de Microbiología, Universidad de Oviedo. Avda. Julián Clavería, 7, 33006 Oviedo, Spain; 2 IUOPA (Instituto Universitario de Oncología del Principado de Asturias), Oviedo, Spain; 3 ISPA (Instituto de Investigación Sanitaria del Principado de Asturias), Oviedo, Spain; 4 El Hórreo Healthy Foods SL. Polígono de Granda 17, 33199 Siero, Spain; 5 COSFER SA, C/Isaac Peral 2, Can Castells, 08420 Canovelles, Barcelona Spain; 6 Geisel School of Medicine at Dartmouth, Dartmouth College, Hannover, NH 03755 New Hampshire, USA; 7 Molecular Histopathology Unit in Animal Models for Cancer, Instituto Universitario de Oncología del Principado de Asturias (IUOPA), Universidad de Oviedo, Oviedo, Spain

**Keywords:** Cancer prevention, Metagenomics

## Abstract

Inulin-rich foods exert a prebiotic effect, as this polysaccharide is able to enhance beneficial colon microbiota populations, giving rise to the *in situ* production of short-chain fatty acids (SCFAs) such as propionic and butyric acids. These SCFAs are potent preventive agents against colorectal cancer due to their histone deacetylases inhibitory properties, which induce apoptosis in tumor colonocytes. As colorectal cancer is the fourth most common neoplasia in Europe with 28.2 new cases per 100,000 inhabitants, a cost-effective preventive strategy has been tested in this work by redesigning common porcine meat products (*chorizo* sausages and cooked ham) consumed by a substantial proportion of the population towards potential colorectal cancer preventive functional foods. In order to test the preventive effect of these inulin-rich meat products against colorectal cancer, an animal model (*Rattus norvegicus* F344) was used, involving two doses of azoxymethane (10 mg/kg) and two treatments with dextran sodium sulfate (DSS) during a 20-week assay period. Control feed, control sausages, functional sausages (15.7% inulin), control cooked ham and functional cooked ham (10% inulin) were used to feed the corresponding animal cohorts. Then, the animals were sacrificed and their digestive tract tissues were analyzed. The results showed a statistically significant 49% reduction in the number of colon polyps in the functional meat products cohorts with respect to the control meat products animals, as well as an increase in the cecum weight (an indicator of a diet rich in prebiotic fiber), a 51.8% increase in colon propionate production, a 39.1% increase in colon butyrate concentrations, and a reduction in the number of hyperplastic Peyer’s patches. Metagenomics studies also demonstrated colon microbiota differences, revealing a significant increase in *Bacteroidetes* populations in the functional meat products (mainly due to an increase in *Bacteroidaceae* and *Prevotellaceae* families, which include prominent propionate producers), together with a reduction in *Firmicutes* (especially due to lower *Lachnospiraceae* populations). However, functional meat products showed a remarkable increase in the anti-inflammatory and fiber-fermentative *Blautia* genus, which belongs to this *Lachnospiraceae* family. The functional meat products cohorts also presented a reduction in important pro-inflammatory bacterial populations, such as those of the genus *Desulfovibrio* and *Bilophila*. These results were corroborated in a genetic animal model of CRC (F344/NSlc-Apc^1588/kyo^) that produced similar results. Therefore, processed meat products can be redesigned towards functional prebiotic foods of interest as a cost-effective dietary strategy for preventing colorectal cancer in human populations.

## Introduction

Colorectal cancer (CRC) is the most common cancer in Western countries (35 cases per 100,000 inhabitants) and represents one of the leading causes of death^1^. The colon mucosa is covered with numerous tube-shaped invaginations called crypts. At the bottom of these crypts, stem cell division takes place^2^. During the cellular division processes in these stem cells, mutations can occur, generating offspring cells with uncontrolled growth that will trigger colon tumor formation. A first stage in this process is the formation of an aberrant crypt (ACF), which can then evolve into a microadenoma and a large adenoma (polyp) to finally generate, after several decades of development, an adenocarcinoma with metastatic capacity (colorectal carcinoma, CRC). This long process causes a higher CRC incidence in countries with aging populations^3,4^.

The main risk factors contributing to these neoplasms are a high consumption of red meat, processed meat (which may include carcinogenic chemicals such as N-nitroso-compounds and polycyclic aromatic hydrocarbons) and saturated fat, among others such as tobacco use and alcohol intake^1^. To further understand these risk factors, a study by the IARC (International Agency for Research on Cancer) analyzed more than 800 epidemiological dossiers that investigated the association of cancer with the consumption of red meat or processed meat in many countries. The conclusion was that a diet including 100 g/day of red meat or 50 g/day of processed meat can lead to a 17–18% increase in the risk of developing cancer, respectively^5^. These types of meat products are common in Western diets, which usually include cured meats and processed products such as sausages^6,7^. By contrast, numerous epidemiological studies found a lower incidence of CRC in human populations with a high consumption of fruits and vegetables (which provide prebiotic fibers that, once fermented in the colon, give rise to short-chain fatty acids, SCFAs) and low consumption of red meat and saturated fat^8^.

These CRC tumorigenic processes in the colon mucosa (from the initial ACF to metastatic adenocarcinoma) can be altered or even stopped by the presence of some nutraceutical compounds in the colon lumen, such as prebiotic fibers. Prebiotics have been defined as *selectively fermented ingredients that result in specific changes in the composition and/or activity of the gastrointestinal microbiota, thus conferring benefits upon host health*^9,10^. The most widespread prebiotic compounds in nature are plant polysaccharides. In plants, most common polysaccharidic energy reserves are usually in the form of starch (D-glucose chains, via α(1–4) glycosidic bonds, with α(1–6) branches). Once ingested by mammals in the form of cereals, potatoes and other food sources, this starch is readily digested, initially in the mouth by the action of amylases, and then by pancreatic and other intestinal enzymes, giving rise to glucose molecules which are absorbed in the small intestine^11,12^. However, in about 10% of plant species, such us garlic, onion, artichoke, asparagus and chicory, and in a few bacterial species, the main polysaccharidic energy reserve is not based on D-glucose chains but on D-fructose chains (via β(1–2) glycosidic bonds), and these types of polymers are called fructans instead of starches. Fructans usually contain 2 to 60 fructoses (sometimes with an initial D-glucose moiety), and are then called fructooligosaccharides (low MW chains) or inulin (over 10 D-fructose moieties)^13–16^.

Unlike starches, fructans such as inulin are not altered in the digestive tract of humans until they reach the colon, where they are used as a source of energy and carbon by the probiotic bacteria of the intestinal microbiota. Among these bacteria are lactic acid bacteria of the genera *Bifidobacterium* and *Lactobacillus* and other SCFAs producing species as a result of the fermentation of these prebiotic polysaccharides^17,18^.

These SCFAs are mainly lactate, pyruvate and acetate, which are used by other colon bacteria as metabolic precursors for propionate and butyrate production^19,20^. Butyrate is the most important SCFA in human health, as it is the preferred energy source of colonocytes and it possesses anti-inflammatory properties. SCFAs also induce differentiation and apoptosis in colonocytes^21^. In terms of energy metabolism, butyrate is the main energy source for normal colonocytes. At low concentrations and under reduced cytoplasmic levels of glucose and pyruvate (as in the crypts cells), butyrate acts as an energy source for normal colonocytes, where it is rapidly processed by β-oxidation in the mitochondria^22^. Dietary intake of inulin or other prebiotic fibers has been associated with a prevention or a reduction in the incidence of colon polyps and tumors, in the presence of bacteria able to ferment these polysaccharides, due to the generation of antitumor SCFAs^23–25^.

In this work, the advantageous effect of prebiotic fibers are applied to two traditional processed meat products: smoked fermented sausages (*chorizo*, widely consumed in Spain and similar versions in other Mediterranean countries) and cooked ham (widely consumed as sliced ham in most Western countries). Both food products are of porcine origin and are listed as foods with a recommended ingestion limit due to their saturated fat and salt content. These food products have been redesigned by, in the case of the smoked fermented sausage, substituting fat with the prebiotic inulin or, in the case of cooked ham, directly adding inulin to the food matrix, eliminating in both cases the use of food chemical additives. These novel prebiotic food products have been tested in two animal models. In both cases rat models for CRC were used as they are more comparable to human processes. The rat colon is larger than a mouse’s and allows for easier observation of tumor processes and the rat’s chromosomal structure is similar to that of humans (metacentric), as opposed to the telocentric chromosomes in mice^26^. The first implemented a fast animal model for CRC studies (male *Rattus norvegicus* Fisher 344), which develops colon polyps in only 20 weeks due to the double effect of the carcinogen azoxymethane (AOM) and the ulcerative colitis inducer dextran sodium sulfate (DSS). The functional prebiotic meat products have been compared with control rat feed and with the corresponding control meat products. Histological, serum and metagenomics parameters have been analyzed in all animal cohorts.

The second animal model for CRC was implemented in a similar way but, in this case, based on a genetic mutation in *apc* gene, which causes early development of colon polyps in these rats, as in human familiar adenomatous polyposis syndrome. The *apc* gene codes for a multifunctional protein (dimerization domain, ASEF binding domain, β-catenin binding domain, β-catenin degradation domain, axin binding domain, microtubule binding domain, and EB1 and HDLG binding domains)^27^. Here, the mutant rat model used was the Kyoto Apc Delta rat (KAD rat), which contains a homozygous mutation in the 2523 codon of *apc* gene, generating a truncated Apc protein (lacking EB1 and hDLG binding domains)^28^. This mutant rat can survive 1.5 years before developing colon polyps, and for this reason it is necessary to induce colon inflammation^29^.

This genetic animal model for CRC has confirmed the preventive effect of the functional meat products redesigned in this work. This indicates that the reformulation of processed red meat food matrixes towards prebiotic meat products is a possible way to provide human populations with easy and cost-effective access to dietary factors involved in CRC prevention, such as inulin, which are not usually present in meat products as they are from plant origin.

## Results

### Effect of functional meat food on serum triacylglycerides levels and body weight

In the case of the chemically induced CRC animal model, animals from the three cohorts (feed, control meat and functional meat) did not show alterations in body weight gain during the 20 experimental weeks, with no differences between cohorts, although this weight gain diminished transitorily during both DSS challenges. After sacrifice, the mean body weight values were 368.6 ± 18.2 g for the feed cohort, 332.5 ± 36.7 g for the control meat cohort, and 329.7 ± 20.2 g for the functional feed cohort. The serum triacylglycerides levels in the functional food cohort (110 ± 5.7 mg/dL) were lower than in the feed cohort (172.1 ± 22.1 mg/dL) to a statistically significant degree (p-value 0.007), but with no differences with respect to the control meat cohort (147 ± 13.2 mg/dL, p-value 0.063). Nevertheless, it is worth mentioning that four animals died during this assay: one from the feed cohort (rat F10), two from the control meat cohort (rats CCH7 and CCH8), and one from the functional meat cohort (rat FS7). Animals F10 and CCH7 died during the first week at the animal facilities, probably due to inherited problems. The two other animals did not survive the first DSS challenge, which was used to enhance the final production and size of the colon tumors. The cause of death was intense rectal bleeding associated with acute ulcerative colitis induced by DSS.

**Figure 1:**
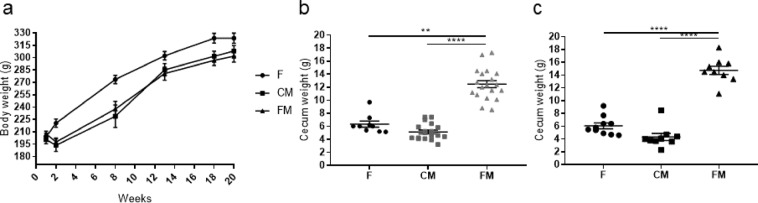
(**a**) Mean body weight for the genetic animal model along the experimental weeks. **(b)** Mean cecum weight in grams for each cohort (only rats 1 to 8 in each group) in the chemically induced CRC animal model. **(c)** Mean cecum weight in grams for each cohort (only rats 1 to 8 in each group) in the genetic CRC animal model. F: feed cohort, CM: control meat cohort, FM: functional meat cohort.

In the genetic CRC animal model, again, animals from the three cohorts showed a continuous and similar weight gain throughout the 20 weeks, although these mutant animals took longer to adapt to the meat diets and, therefore, the two meat cohorts suffered a small loss in body weight during the first two weeks of the assay. As in the previous assay, one rat from the functional meat cohort (rat FM7) died during the DSS challenge at week 4 due to intense rectal bleeding. After sacrifice, the mean body weight values were 323.7 ± 6.1 g for the feed cohort, 308 ± 6.5 g for the control meat cohort, and 301.7 ± 7.1 g for the functional feed cohort (Fig. 1a).

### Effect of functional meat food on cecum weight

In the chemically induced CRC animal model, there were statistically significant differences in the cecum weight values between the functional meat cohort (12.4 ± 0.5 g) and the feed cohort (6.3 ± 0.4 g, p-value < 0.002), as well as between the functional meat cohort and the control meat cohort (5,1 ± 0,2 g, p-value < 0.0001) (Fig. 1b).

In the case of the genetic CRC animal model, there were statistically significant differences in the cecum weight values between the functional meat cohort (14,7 ± 0,6 g) and the feed cohort (6,0 ± 0,4 g, p-value < 0.001), as well as between the functional meat cohort and the control meat cohort (4,3 ± 0,5 g, p-value < 0.001) (Fig. 1c). The reason for the increased cecum weight in the functional meat animals is therefore due to the increased growth of bacterial populations as a result of the presence of the prebiotic (fermentable) fiber inulin in these food matrixes.

### Effect of functional meat food on hyperplastic Peyer’s patches

After sacrifice, the hyperplastic Peyer’s patches were quantified along the small intestine. These lymphoid tissues contain a multitude of lymphocytes that can become hyperplastic, showing a rounded, protruding, white 2–3 mm ovals aspect^30^.

**Figure 2:**
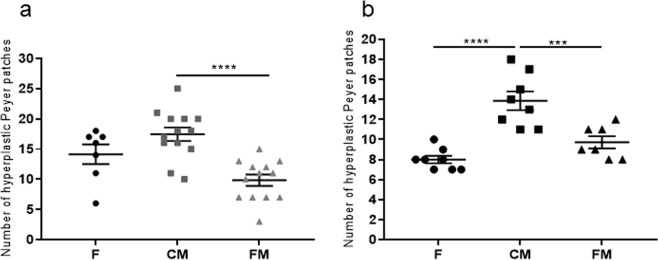
(**a**) Mean number of hyperplastic Peyer’s patches in the small intestines from each cohort (rats 1 to 8 in the three groups) in the chemically induced CRC animal model. (**b**) Mean number of hyperplastic Peyer’s patches in the small intestines from each cohort (rats 1 to 8 in the three groups) in the genetic CRC animal model. F: feed cohort, CM: control meat cohort, FM: functional meat cohort.

In the chemically induced CRC animal model, there were statistically significant differences in the mean value for Peyer’s patches only between the functional meat cohort (9.8 ± 0.9) and the control meat cohort (17.4 ± 1.1, p-value < 0.0001). The feed cohort animals showed an average of 14.1 ± 1.6 patches (Fig. 2a).

In the case of the genetic CRC animal model, there were statistically significant differences in the mean value for Peyer’s patches between the functional meat cohort (9.7 ± 0.6) and the control meat cohort (13.8 ± 0.9, p-value < 0.001), but also between the control meat cohort and the feed cohort (8 ± 0.3, p-value < 0.0001) (Fig. 2b).

### Effect of functional meat food on the number of polyps and total polyp-affected area

**Figure 3:**
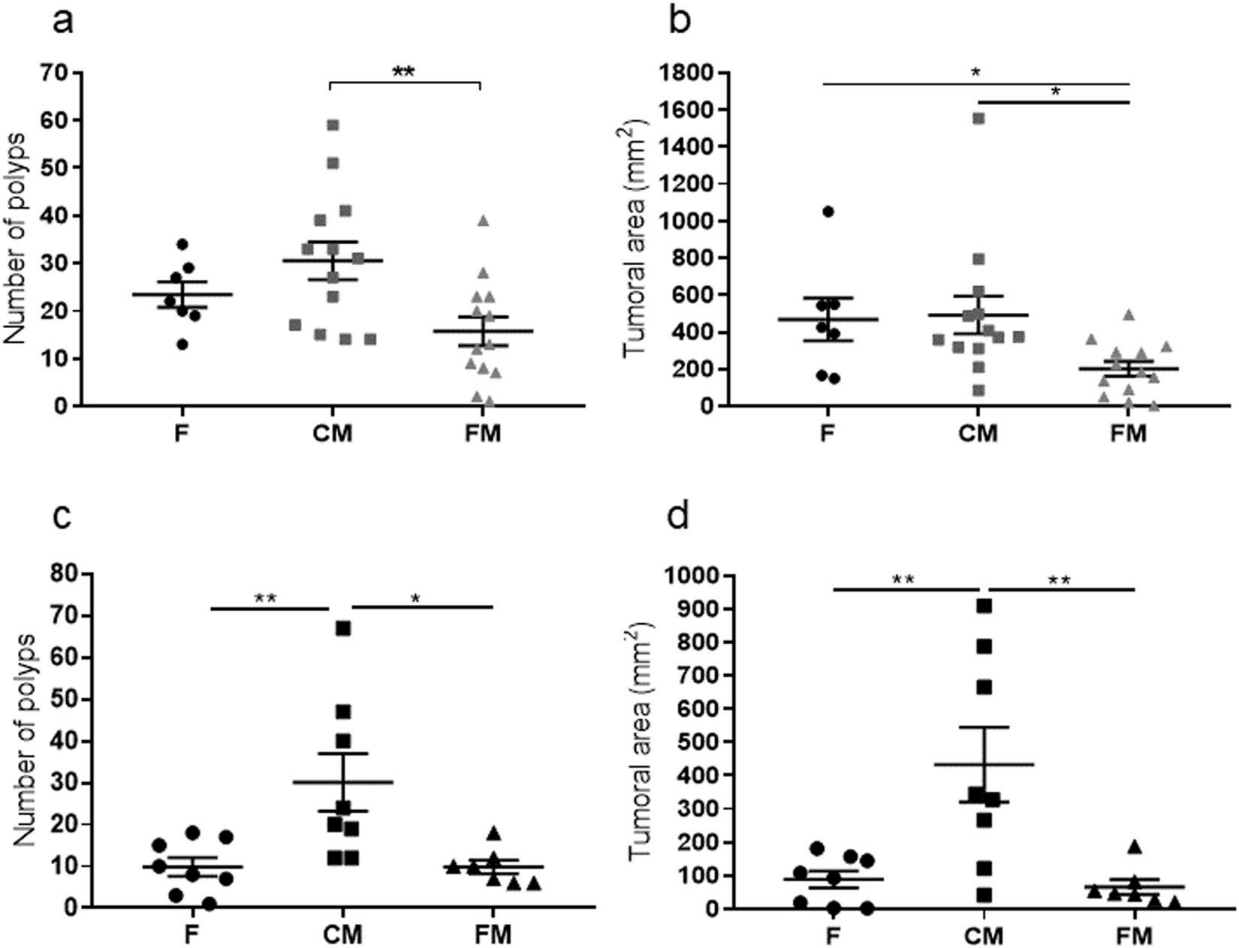
(**a**) Mean number of colon polyps from each cohort in the chemically induced CRC animal model (rats 1 to 8 in the three groups). The absolute control rats showed zero colon polyps (rats 9 and 10 from each cohort). (**b**) Mean value in mm^2^ of the sum of polyp areas from all polyps in all animals from each cohort (rats 1 to 8 in the three groups) in the chemically induced CRC animal model. (**c**) Mean number of colon polyps from each cohort in the genetic CRC animal model (rats 1 to 8 in the three groups). The absolute control rats showed zero colon polyps (rats 9 and 10 from each cohort). (**d**) Mean value in mm^2^ of the sum of polyp areas from all polyps in all animals from each cohort (rats 1 to 8 in the three groups) in the genetic CRC animal model. F: feed cohort, CM: control meat cohort, FM: functional meat cohort.

**Figure 4:**
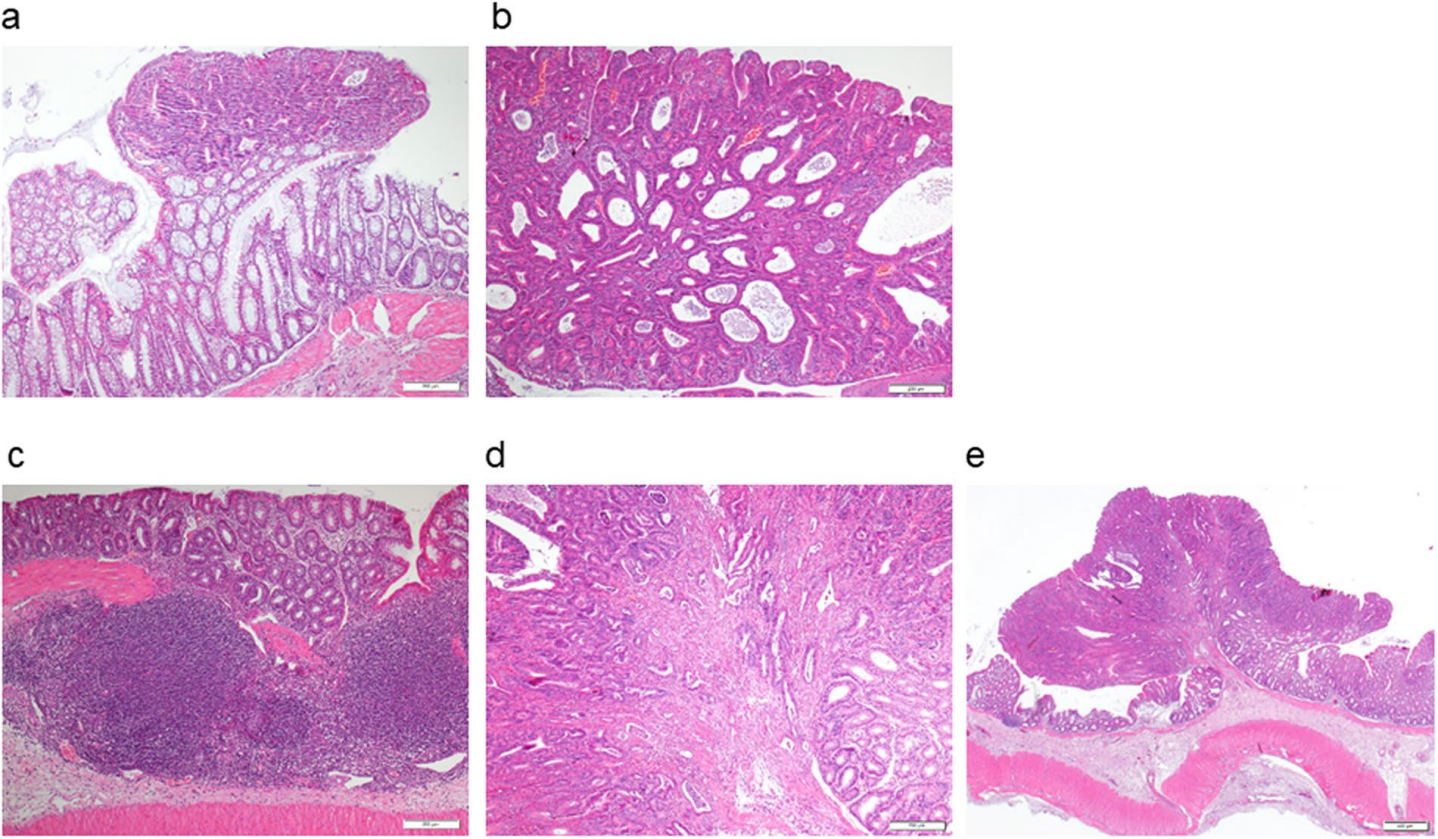
Optical microscope images of sections from colon polyps after hematoxylin and eosin staining. (**a**) Example of tubular adenoma with low degree dysplasia (4x). (**b**) Example of tubular adenoma with high degree dysplasia (4x). (**c**) Example of lymphoid hyperplasia (4x). (**d**) Example of infiltrating adenocarcinoma (4x). (**e**) Example of infiltrating adenocarcinoma (1.25x).

After sacrifice, colon mucosae were analyzed for the number of polyps, which ranged from 1 mm to 10 mm in diameter. In the chemically induced CRC animal model, a statistically significant difference was only observed between rats of the control meat cohort (30.5 ± 3.9) and functional meat cohort (15.3 ± 3, p-value < 0.008), which experienced a 49.9% reduction in the number of polyps (Fig. 3a). The number of polyps in the feed cohort was 23.4 ± 2.3, slightly higher than in the functional meat cohort but lacking statistical significance. Also, the area of each polyp present in a given colon mucosa was calculated according to its shape and the total polyp area was computed for each animal. A statistically significant reduction (56.9%) in the total polyp-affected area was observed in the functional meat cohort (201,4 ± 40,5 mm^2^) with respect to feed cohort (467,7 ± 114,7 mm^2^, p-value < 0.04) and to the control meat cohort (491,4 ± 101,3 mm^2^, p-value < 0.01) (Fig. 3b). Notably, histological studies on these polyps indicated a reduction in the number of low degree dysplasias in the functional meat colons with respect to the other two cohorts; and only two infiltrating adenocarcinomas were detected, one in the feed cohort, and another one in the control meat cohort (Fig. 4).

In the genetic CRC animal model, a statistically significant reduction in the number of polyps was observed in the functional meat cohort (9.8 ± 1.6, p-value < 0.01) and the feed cohort (9.8 ± 6.3, p-value < 0.009), both with respect to the control meat cohort (30.1 ± 6.8) (Fig. 3c). As for the total polyp-affected area, statistically significant differences were found in the functional meat cohort (65.8 ± 21.7 mm^2^, p-value < 0.003) and the feed cohort (88.5 ± 25.5 mm^2^, p-value < 0.003), both with respect to the control meat cohort (433.1 ± 112.2 mm^2^) (Fig. 3d).

### Effect of functional meat food on the cecum concentrations of SCFAs

**Figure 5:**
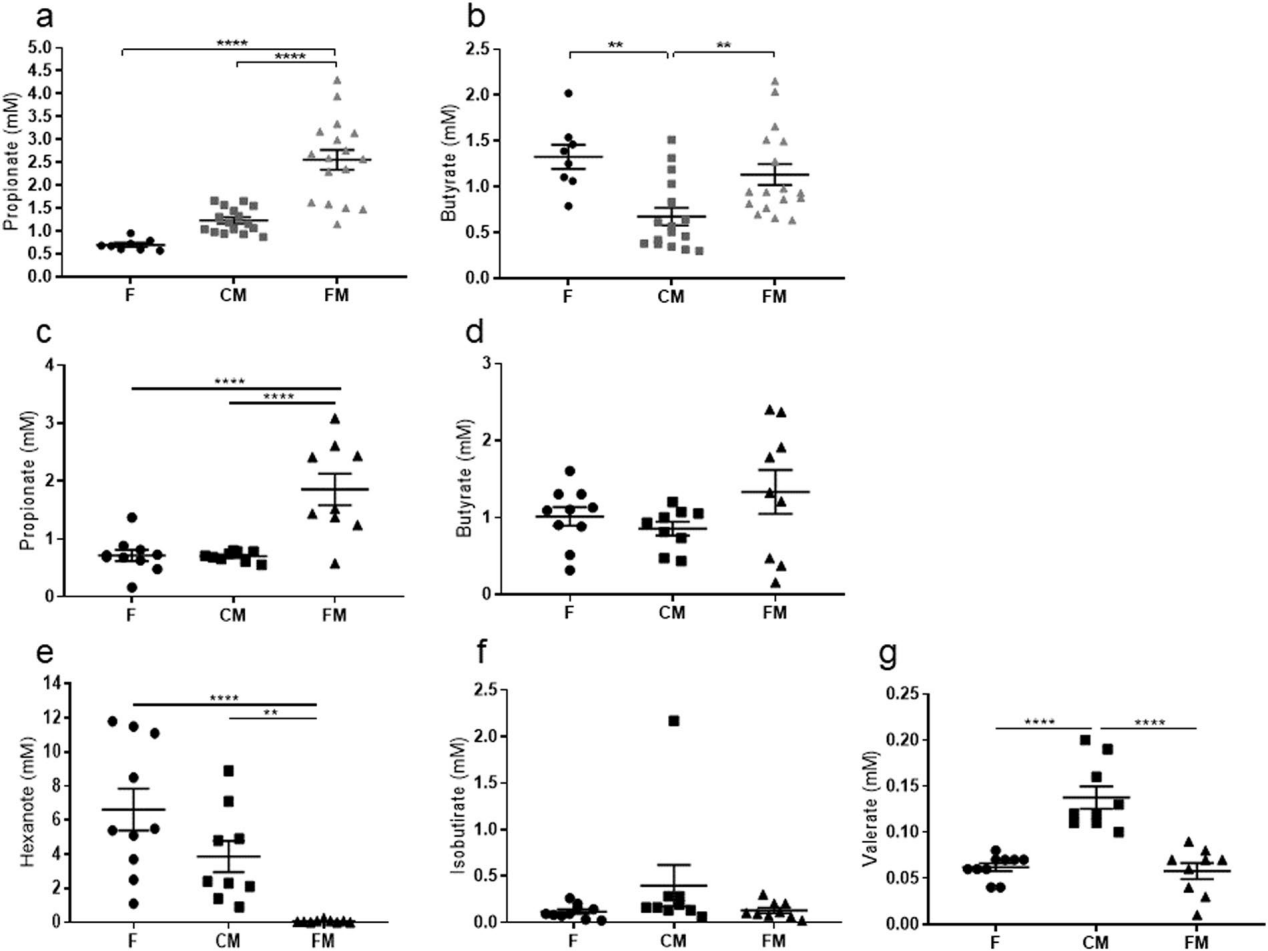
Mean SCFA concentrations in cecum feces. (**a**) Propionate, and (**b**) butyrate concentrations in each cohort in the chemically induced CRC animal model, (**c**) Propionate, (**d**) butyrate, (**e**) hexanoate, (**f**) isobutyrate and (**g**) valerate concentrations in each cohort in the genetic CRC animal model. F: feed cohort, CM: control meat cohort, FM: functional meat cohort. (N = 8).

Feces were taken from the cecum in order to quantify the SCFAs concentrations by GC-MS using isotopic internal standards (see Materials and Methods section). In the chemically induced CRC animal model, statistically significant differences were only observed in the case of propionate and butyrate. Cecum propionate concentrations were 72.6% higher in the functional meat cohort (2.5 ± 0.2 mM) with respect to the feed cohort (0.69 ± 0.04 mM, p-value < 0.0001), and also 51.8% higher with respect to the control meat cohort (1.23 ± 0.06 mM, p-value 0.0001) (Fig. 5a).

Cecum butyrate concentrations in this case were lower in the control meat cohort (0.67 ± 0.09 mM) to a statistically significant degree with respect to both the feed cohort (1.32 ± 0.13 mM, p-value < 0.002) and the functional meat cohort (1.1 ± 0.11 mM, p-value < 0.009) (Fig. 5b).

In the genetic CRC animal model, a statistically significant difference was observed in the case of propionate, hexanoate, isobutyrate and valerate. The production levels of cecum propionate were higher in the functional meat cohort (1.8 ± 0.2 mM) with respect to both the feed cohort (0.7 ± 0.09 mM, p-value < 0.0001) and the control meat cohort (0.7 ± 0.02 mM, p-value < 0.0001) (Fig. 5c). The concentrations of cecum butyrate, though lacking statistical significance, were 1 ± 0.1 mM in the feed cohort, 0.8 ± 0.08 mM in the control meat cohort and 1.3 ± 0.2 mM in the functional meat cohort (Fig. 5d). A statistically significant difference was observed for hexanoate in the functional meat cohort (0.06 ± 0.02 mM) with respect to both the feed cohort (6.6 ± 1.2 mM, p-value < 0.0001) and the control meat cohort (3.8 ± 0.9 mM, p-value < 0.005) (Fig. 5e). As for isobutyrate, no statistically significant differences were observed: 0.1 ± 0.02 mM in the feed cohort, 0.4 ± 0.2 mM in the control meat cohort and 0.1 ± 0.02 mM in the functional meat cohort (Fig. 5f). Finally, statistically significant differences of valerate were observed in the control meat cohort (0.1 ± 0.01 mM) with respect to both the feed cohort (0.06 ± 0.004 mM, p-value < 0.0001) and the control meat cohort (0.06 ± 0.008 mM, p-value < 0.0001) (Fig. 5g).

### Effect of functional meat food on cecal microbiota

In the chemically induced CRC animal model, the cecal metagenomics showed considerable differences in average phyla compositions between the feed cohort and the two meat cohorts (control and functional) (Table 1). At phylum level, the main distinction observed was the increase in *Bacteroidetes* in the control meat (29.58%) and the functional meat (52.75%) cohorts with respect to the feed cohort (15.97%), both statistically significant, as well as increased *Bacteroidetes* in the functional meat cohort with respect to control meat cohort. Additionally, there were statistically significant reductions in *Firmicutes* in the feed cohort (83.47%) with respect to the control meat cohort (67.10%) or the functional meat cohort (42.49%), though here the differences lack significance between the meat cohorts. Finally, a statistically significant increase was observed in *Proteobacteria* between the titers in the feed cohort (0.29%) and both meat cohorts: the control (2.94%) and the functional (4.69%). Specifically, *Proteobacteria* was almost absent in the rats of the feed cohort, whereas it is the second most common phylum in the control sausage cohort and the third most common phylum in the functional sausage cohort.

**Table 1:** Cecal microbiota composition (percentages) at the phylum level where statistically significant differences were observed in the chemically induced CRC animal model.

Phylum	Feed	Control meat	Functional meat	F-CM	F-FM	CM-FM
*Actinobacteria*	0.06	0.10	0.05			
*Bacteroidetes*	15.97	29.58	52.75	*	****	****
*Deferribacteres*	0.01	0.02	0.00			
*Firmicutes*	83.47	67.10	42.49	**	****	****
*Proteobacteria*	0.29	2.94	4.69	*	***	
*Synergistetes*	0.03	0.17	0.00	**		****
*Tenericutes*	0.11	0.09	0.00			

F: feed cohort, CM: control meat cohort, FM: functional meat cohort. * indicate statistically significant differences between each pair of compared cohorts.

In the genetic CRC animal model, at the phylum level there was also an increase in *Bacteroidetes* in the control meat cohort (48.38%) with respect to feed cohort (25.71%). However, no statistically significant difference was observed in the case of the functional meat cohort (26.96%) for this phylum. Similarly, *Firmicutes* titers showed a statistically significant reduction in the case of the functional meat cohort (32.10%) with respect to the two other cohorts, feed (69.61%) and control meat (61.59%). Finally, a statistically significant difference was observed in *Proteobacteria* populations between all three cohorts: 2.26% in the feed cohort, 9.72% in the control meat cohort, 17.79% in the functional meat cohort. There was a total absence of *Synergistetes* and *Tenericutes* in the functional meat cohort, whereas these phyla were present in feed cohort (0.15% and 0.68% respectively) and in the control meat cohort (0.05% and 0.22%).

**Figure 6:**
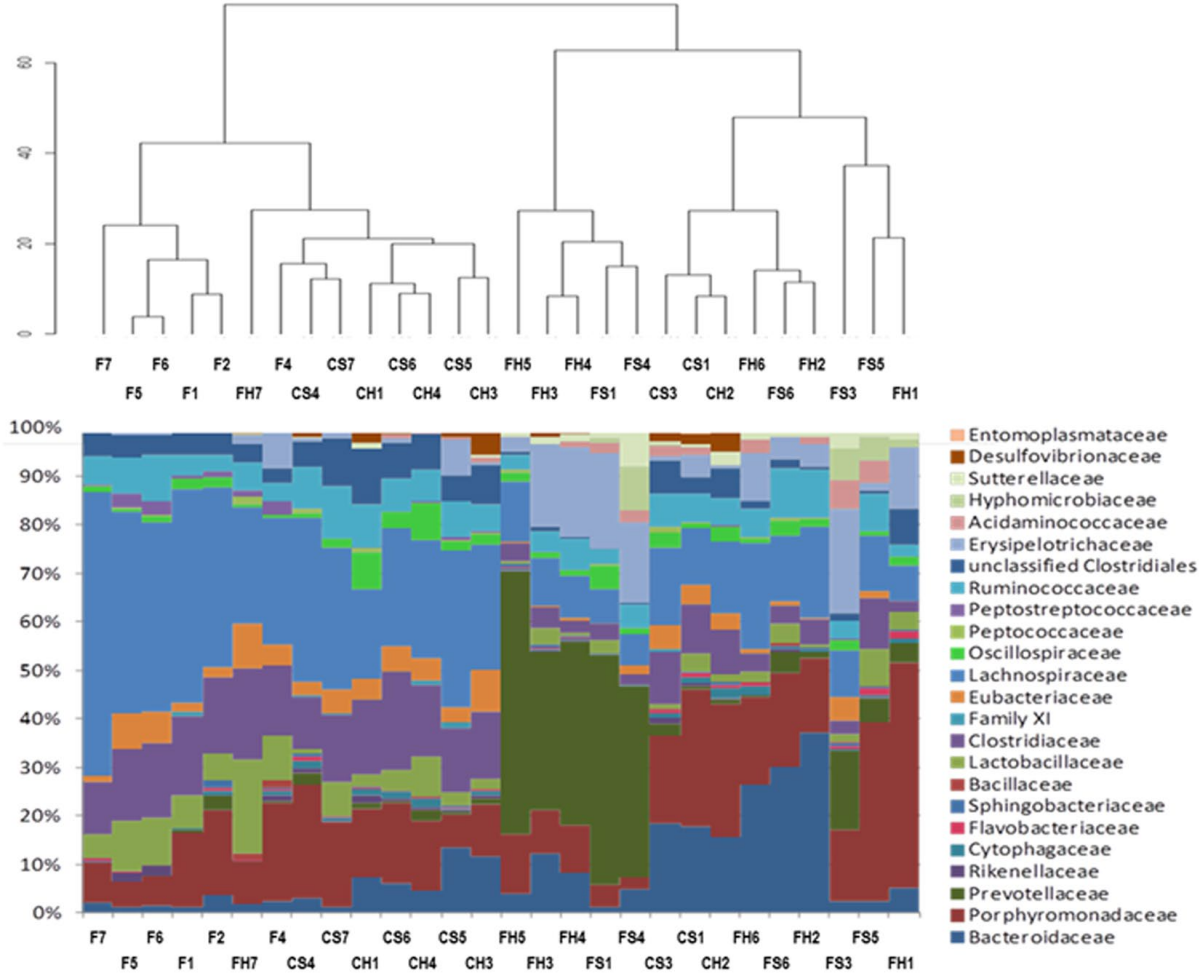
Differences in cecal microbiota composition at the family level for the rats belonging to the three cohorts in the chemically induced CRC animal model. F: feed, CH: control ham, CS: control sausage; FH: functional ham; FS: functional sausage.

At the family level, in the chemically induced CRC animal model, there was a homogenous distribution of the cecum microbiota profile among the animals in each cohort (Fig. 6). The feed cohort animals (51.9% *Lachnospiraceae*, 13.9% *Clostridiaceae*, 9.6% *Porphyromonadaceae*) clustered together with the control meat cohort (31.9% *Lachnospiraceae*, 13.6% *Clostridiaceae*, 13.2% *Porphyromonadaceae*, 12.5% *Bacteroidaceae*), and the main families were similar in both cohorts (Fig. 6). Interestingly, the functional meat cohort animals showed a different profile at the family level, and here the most abundant families were *Prevotellaceae* (22.8%), *Bacteroidaceae* (15.8%), *Porphyromonadaceae* (12%), *Lachnospiraceae* (11.9%) and *Erysipelotrichaceae* (11.3%) (Fig. 6). These differences were statistically significant between the functional meat cohort and the other two.

In the genetic CRC animal model, the cecal microbiota families showed similar characteristics. The main families in the feed cohort animals were again *Lachnospiraceae* (30.3%), *Clostridiaceae* (15.2%) and *Porphyromonadaceae* (15.1%), as in the case of the control meat cohort (25.9% *Lachnospiraceae*, 13.8% *Porphyromonadaceae* and 12.5% *Clostridiaceae*). And the most abundant families in the functional meat cohort, as in the previous case, showed statistically significant differences: *Porphyromonadaceae* (20,7%), *Prevotellaceae* (12.3%), *Desulfovibrionaceae* (9.5%), *Bacteroidaceae* (8.1%), *Lachnospiraceae* (8.1%), and *Sutterellaceae* (7.9%) (Fig. 6).

Tables 2 and 3 present the percent abundance of the genera and species with statistically significant differences between the three cohorts in the chemically induced and genetic CRC animal models. In the first model, the main differences are associated with a higher proportion of some genera in the functional meat cohort (*Bacteroides*, *Blautia*, *Phascolarctobacterium*, *Gemmiger* and *Parasutterella*), and a reduction in the populations of other genera in this functional cohort (*Lactobacillus*, *Clostridium* and *Ruminococcus*). Table 3 indicates the main species involved. In the genetic CRC animal model, the functional meat cohort showed a statistically significant increase in the genera *Anaerostipes, Blautia, Dorea, Lachnoclostridium, Faecalibacterium, Flavonifractor, Eubacterium, Parasutterella* and *Bilophila*, and a reduction in *Prevotella*, *Lactobacillus*, *Clostridium*, *Ruminococcus*, *Turicibacter* and *Desulfovibrio* (Table 4).

**Table 2:** Cecal microbiota composition (percentages) at the phylum level where statistically significant differences were observed in the genetic CRC animal model. QIIME-2 software was used for these studies.

Phylum	Feed	Control meat	Functional meat	F-CM	F-FM	CM-FM
*Actinobacteria*	0.52	0.48	0.69			
*Bacteroidetes*	25.71	26.96	48.38		****	****
*Firmicutes*	69.61	61.59	32.10		****	****
*Proteobacteria*	2.26	9.72	17.79	**	****	***
*Deferribacteres*	0.34	0.42	0.20			
*Lentisphaerae*	0.03	0.00	0.01			
*Spirochaetes*	0.00	0.14	0.00			
*Synergistetes*	0.15	0.05	0.00	*	****	
*Tenericutes*	0.68	0.22	0.00		***	*
Unclassified bacteria	0.16	0.11	0.00			
*Verrucomicrobia*	0.10	0.01	0.23			

F: feed cohort, CM: control meat cohort, FM: functional meat cohort. * indicate statistically significant differences between each pair of compared cohorts.

**Table 3:** Cecal microbiota composition (percentages) at the genus and species levels where statistically significant differences were observed in the chemically induced CRC animal model.

Genera	Species	Feed	Control meat	Functional meat	F-CM	F-FM	CM-FM
*Bacteroides*	*spp*.	0.610	8.039	11.339	**	**	
	*caccae*	0.000	0.803	0.734	**	**	
	*thetaiotaomicron*	0.000	0.104	0.105	*	*	
	*vulgatus*	0.237	2.798	0.641	*		
*Parabacteroides*	*spp*.	0.203	1.095	1.161		*	
	*merdae*	0.063	0.509	0.608	*	**	
*Lactobacillus*	*spp*.	7.310	4.436	2.383	*		
	*intestinalis*	1.612	0.270	0.196	**	*	
	*reuteri*	0.205	0.064	0.011	*	**	
	*taiwanensis*	0.765	0.000	0.000	****	****	
	*vaginalis*	1.023	0.324	0.064	*	**	
*Clostridium*	*spp*.	5.498	6.075	3.463			**
	*hiranonis*	0.135	0.027	0.000	*	***	
*Anaerostipes*	*spp*.	0.007	1.975	0.783	**		
*Blautia*	*spp*.	0.000	3.863	5.382	*	**	
	*glucerasea*	0.000	0.071	0.386		**	*
	*producta*	0.000	0.909	0.387	*		
*Dorea*	*spp*.	0.000	0.516	0.115	*		
*Eubacterium*	*spp*.	0.000	0.495	0.000			*
	*hadrum*	0.000	0.495	0.000			*
*Lachnoanaerobaculum*	*spp*.	0.173	0.118	0.000		**	
*Marvinbryantia*	*spp*.	0.000	0.185	0.052	*		
*Ruminococcus*	*gnavus*	0.435	0.291	0.000		***	*
*(Ruminococcus)*	*spp*.	19.462	9.238	0.223		***	*
	*gnavus*	6.722	6.082	0.062		***	**
*Acetanaerobacterium*	*spp*.	0.037	0.124	0.000			*
*Ruminococcus*	*flavefaciens*	0.548	0.018	0.000	*	*	
*Flavonifractor*	*spp*.	0.000	0.326	0.820	*	*	
	*plautii*	0.000	0.235	0.622	*	*	
*(Eubacterium)*	*spp*.	0.000	0.084	0.484		*	
	*dolichum*	0.000	0.084	0.484		*	
*Phascolarctobacterium*	*spp*.	0.000	0.590	1.821		*	
	*succinatutens*	0.000	0.512	1.651		*	
*Gemmiger*	*spp*.	0.000	0.000	1.702		*	**
	*formicilis*	0.000	0.000	1.702		*	**
*Parasutterella*	*spp*.	0.012	0.584	1.863		***	*
	*excrementihominis*	0.005	0.494	1.743		***	*
*Bilophila*	*spp*.	0.053	1.275	0.365	*		
	*wadsworthia*	0.045	0.924	0.273	*		
*Desulfovibrio*	*spp*.	0.218	0.880	0.252	**		***
	*piger*	0.103	0.762	0.231	***		**

F: feed cohort, CM: control meat cohort, FM: functional meat cohort. * indicate statistically significant differences between each pair of compared cohorts.

**Table 4:** Cecal microbiota composition (percentages) at the genus and species levels where statistically significant differences were observed in the genetic CRC animal model.

Genera	Species	F	CM	FM	F-CM	F-FM	CM-FM
*Bacteroides*	*spp*.	0.869	3.217	1.526	*		
	*acidifaciens*	0.484	2.568	0.017		*	****
	*thetaiotaomicron*	0.000	0.043	0.076		**	
*Prevotella*	*spp*.	0.644	0.606	0.104		*	
*Alistipes*	*shahii*	0.000	0.042	0.090		*	
*Lactobacillus*	*intestinalis*	2.439	0.113	0.122	***	***	
	*johnsonii*	0.732	0.162	0.018	**	***	
	*reuteri*	0.389	0.448	0.006		**	
	*vaginalis*	1.229	1.134	0.034		***	**
*Clostridium*	*spp*.	7.984	5.877	4.522		*	
	*bolteae*	0.000	0.000	0.149		**	**
	*vincentii*	0.094	0.011	0.000	*	**	
*Anaerostipes*	*caccae*	0.000	0.000	0.079		*	*
*Blautia*	*spp*.	0.106	0.558	0.959		*	
	*wexlerae*	0.000	0.000	0.149		***	***
	*glucerasea*	0.004	0.218	0.000	*		**
	*producta*	0.000	0.029	0.058		*	
*Coprococcus*	*spp*.	0.187	0.021	0.000		**	
*Dorea*	*dorea*	0.000	0.027	0.179		*	
	*formicigenerans*	0.091	0.150	0.000			*
*Lachnoclostridium*	*spp*.	0.132	0.187	1.187		**	*
	*clostridioforme*	0.000	0.000	0.966		***	***
*Marvinbryantia*	*formatexigens*	0.003	0.004	0.059		**	*
*Oribacterium*	*spp*.	0.213	0.029	0.000		**	
*Parasporobacterium*	*spp*.	0.040	0.086	0.006	*		***
*Tyzzerella*	*spp*.	0.119	0.174	0.014		*	**
[*Ruminococcus*]	*spp*.	9.621	8.587	0.543		***	**
	*gnavus*	2.669	3.518	0.407		**	***
*Oscillibacter*	*valericigenes*	0.046	0.086	0.002		*	**
*Faecalibacterium*	*spp*.	1.133	1.850	3.081		**	
*Ruminiclostridium*	spp.	0.143	0.164	0.014		**	***
*Ruminococcus*	*flavefaciens*	0.382	0.059	0.004	**	***	
*Subdoligranulum*	*spp*.	0.000	0.000	0.096		****	****
*Flavonifractor*	*spp*.	0.184	0.056	0.710			*
	*plautii*	0.000	0.000	0.641		**	**
*Pseudoflavonifractor*	*spp*.	0.060	0.286	0.121	*		
*[Eubacterium]*	*dolichum*	0.000	0.000	1.502		***	***
*Holdemania*	*filiformis*	0.000	0.014	0.061		**	
*Turicibacter*	*spp*.	1.066	0.087	0.012	**	****	
*Parasutterella*	spp.	0.039	0.517	7.930		****	****
	*excrementihominis*	0.039	0.486	7.423		****	****
*Bilophila*	*spp*.	0.059	0.832	9.411		****	*
	*wadsworthia*	0.053	0.731	8.083		****	*
*Desulfovibrio*	*spp*.	0.918	0.912	0.129			*
*Akkermansia*	*muciniphila*	0.095	0.006	0.229			*

F: feed cohort, CM: control meat cohort, FM: functional meat cohort. * indicate statistically significant differences between each pair of compared cohorts.

Finally, the Chao diversity indexes were calculated for the different rats’ cohorts in both animal models (wild type and genetic). As can be observed in Fig. 8, in both cases the meat diets (control and functional) showed an increase in the diversity of species found in the cecal microbiota.

## Discussion

The objective of this study was to re-design two common processed meat products, traditional smoked *chorizo* sausages and cooked ham, in order to eventually transform them into functional meat foods of interest in the prevention of CRC, the most widespread cancer type in Western countries^1^.

*Chorizo* is widely consumed in some European, American and Asian countries, such as Spain, Portugal, Argentina, Chile, Paraguay, Uruguay, Bolivia, Peru, Ecuador, Colombia, Brazil, Mexico and Philippines. Sliced cooked ham is frequent in the shopping basket of European, American and Oceanian countries. The new functional versions of both meat products were produced without the addition of preservatives (such as nitrates) and with a very low fat content. This low fat content, in the case of functional *chorizo*, was achieved by replacing bacon (one of the traditional ingredients) with the plant prebiotic polysaccharide inulin. Inulin is widely used in low fat foods, such as chocolate and ice cream, where it mimics some of the organoleptic properties associated with animal fat, but lacks cholesterol and caloric content^14,15^. In this study, inulin replaced most of the *chorizo* fat, and was included in the cooked ham brine solution as well. Final inulin concentrations were 15.7% in chorizo and 10% in cooked ham. Both concentrations were optimized independently in order to not alter the taste and the palatability of the novel functional meat products, as higher concentrations created a food matrix with different palatability to both control meat products due to the presence of inulin.

The reason for the prebiotic properties of the functional meat products developed in this work is that mammal digestive enzymes do not digest inulin (a long fructose polysaccharide), and therefore it arrives intact to the colon. In the colon, specific microbiota populations ferment inulin, giving rise to a plethora of SCFAs (propionate, butyrate, valerate, etc.) with antitumor effects on tumor colonocytes. In CRC cells, glucose is the main energy source, with glucose uptake increased about 10 times due to the overexpression of GLUT transporters and to the Warburg effect. This excess of glucose in CRC cells displaces butyrate as the main energy source, causing butyrate to accumulate in the CRC cell cytoplasm and later in the cell nucleus. This nuclear butyrate accumulation gives rise to histones hyperacetylation, since butyrate is a strong histone deacetylases (HDAC) inhibitor, finally leading to apoptosis induction, which proceeds via the intrinsic/mitochondrial pathway^10,16,31,32^. Histones acetylation is one of the main regulatory mechanisms in modulating genetic expression. This acetylation alters the accessibility to DNA transcription, which is a key issue during tumor formation^33^. Acetylation of histones H3 and H4 neutralizes the positive charges in their L-Lys residues and disrupts the nucleosome structure, enabling DNA unfolding and a more relaxed chromatin structure, which ultimately allows access to transcription factors and the activation of pro-apoptotic gene transcription. So, histone acetylation due to butyrate HDAC inhibitory activity permits the genetic transcription of anticancer factors such as p21^10,18,21,31,34–37^. Also, *chorizo* smoking was carried out with a novel method that prevents the formation of benzopyrenes, a potential diet carcinogen, by using a low temperature wood friction approach (300 °C pyrolysis)^38^.

Serum triacylglycerides showed a statistically significant reduction in the functional meat cohort (110 ± 5.7 mg/dL) with respect to the feed cohort (172.1 ± 22.1 mg/dL), but also a non-significant reduction in comparison with the control meat cohort (147 ± 13.2 mg/dL). This could represent some potential protection against cardiovascular diseases, obesity, diabetes, renal failure and other inflammatory processes^39,40^, as noted in another study where a 20–30% reduction in serum triacylglycerides was associated with the consumption of inulin-containing foods^41^. Therefore, the substitution of saturated fat with inulin in this functional meat contributed to its healthier status, including in this case cardioprotective characteristics.

There was also a statistically significant reduction in the number of hyperplastic Peyer’s patches in the small intestine mucosa of the functional meat cohort with respect to the control meat cohort in both animal models (Fig. 2a,b). These mucosa structures are rich in T-lymphocytes and become hyperplastic when alterations in the digestive tract affecting the animal’s immune condition take place, as in response to some chemicals, toxins or pathogens^42,43^. This parameter has been studied as a putative marker of the general condition of the small intestine mucosa status in these animal models, as they suffer hyperplastic alterations under different stress conditions^44,45^. Both functional meat products show, therefore, a protection against the formation of hyperplastic Peyer’s patches, probably due to the fermentation of inulin towards anti-inflammatory SCFAs, such as propionic and butyric acid^21^.

Cecum weight (the whole cecum, including cecum content feces) increased to a statistically significant degree only in the functional meat cohorts with respect to feed and control meat cohorts in both animal models (Fig. 1b,c). This increase corroborated the prebiotic effect of the inulin present in these functional meats. This is because the cecum works as a bioreactor, where microbiota flourish in the presence of fermentable fibers, such as inulin, increasing the weight of this organ and its contents^46,47^.

It is striking that daily administration of the functional meat to these animals throughout the 20 experimental weeks was able to significantly reduce (49.9%) the number of colon polyps (Fig. 3a,c), as well as the total polyp area (59%) in the colon mucosa (Fig. 3b,d), with respect to the control meat cohort. Also, in the case of the chemically induced CRC model, the reduction of total polyp area in the functional meat cohort (56.9%) was statistically significant with respect to the feed cohort (Fig. 3b). Therefore, the inclusion of inulin in the formulation of these meat products has converted them into functional meats of interest in the potential prevention of CRC in human populations. These results are in accordance with other studies where inulin was used to reduce CRC development^48,49^. In both animal models, the greatest number of polyps was observed in the control meat cohort (Fig. 3), since these animals were lacking the protective effect of inulin, and they were also getting half the amount of rat feed (which contains some fermentable fiber) in comparison to feed cohort.

Part of this reduction of colon polyp numbers can be attributed to the marked increase in the production of SCFAs in the cecum of the functional meat cohorts from both animal models, particularly the increase in propionate and butyrate, which are important antitumor SCFAs for CRC prevention^10,50^. Of these, propionate was found to have a greater increase in concentration than butyrate (Fig. 5a,c), with a 72.6% increase with respect to the feed cohort and a 51.8% increase with respect to the control meat cohort. By contrast, since rat feed contains plant fermentable fibers capable of generating butyrate in the rat colon, butyrate concentrations were already higher in the feed cohort. Thus a significant increase in this SCFA in the functional meat cohort was only observed with respect to the control meat cohort (39.1%) (Fig. 5b). Therefore, in these animal models the noted reduction in colon polyp numbers (see above) can be attributed mainly to the observed increase in propionate.

With respect to the cecum microbiota populations, the *Firmicutes/Bacteroidetes* coefficient, sometimes described as a parameter associated with obesity status, was higher in the feed cohort (6.7 ± 1.5) than in the control meat (2.7 ± 0.4, p-value < 0.001) or functional meat (0.8 ± 0.06, p-value < 0.0001) cohorts. The same results appeared in the genetic CRC animal model. The factor responsible for these changes in this coefficient was the increase in the *Bacteroidetes* phylum in the functional meat cohort (67.7% and 43.9% with respect to the feed and the control meat cohorts in the chemically induced CRC animal model, for example). High values for this coefficient (high *Firmicutes* populations) are usually associated with inflammation-associated diseases, such as diabetes or obesity^51,52^. In this work, the higher proportion of *Bacteroidetes* phylum (but also *Proteobacteria*) observed in the inulin functional meat cohort is in accordance with the microbiota results in other studies with inulin^53–55^.

**Figure 7:**
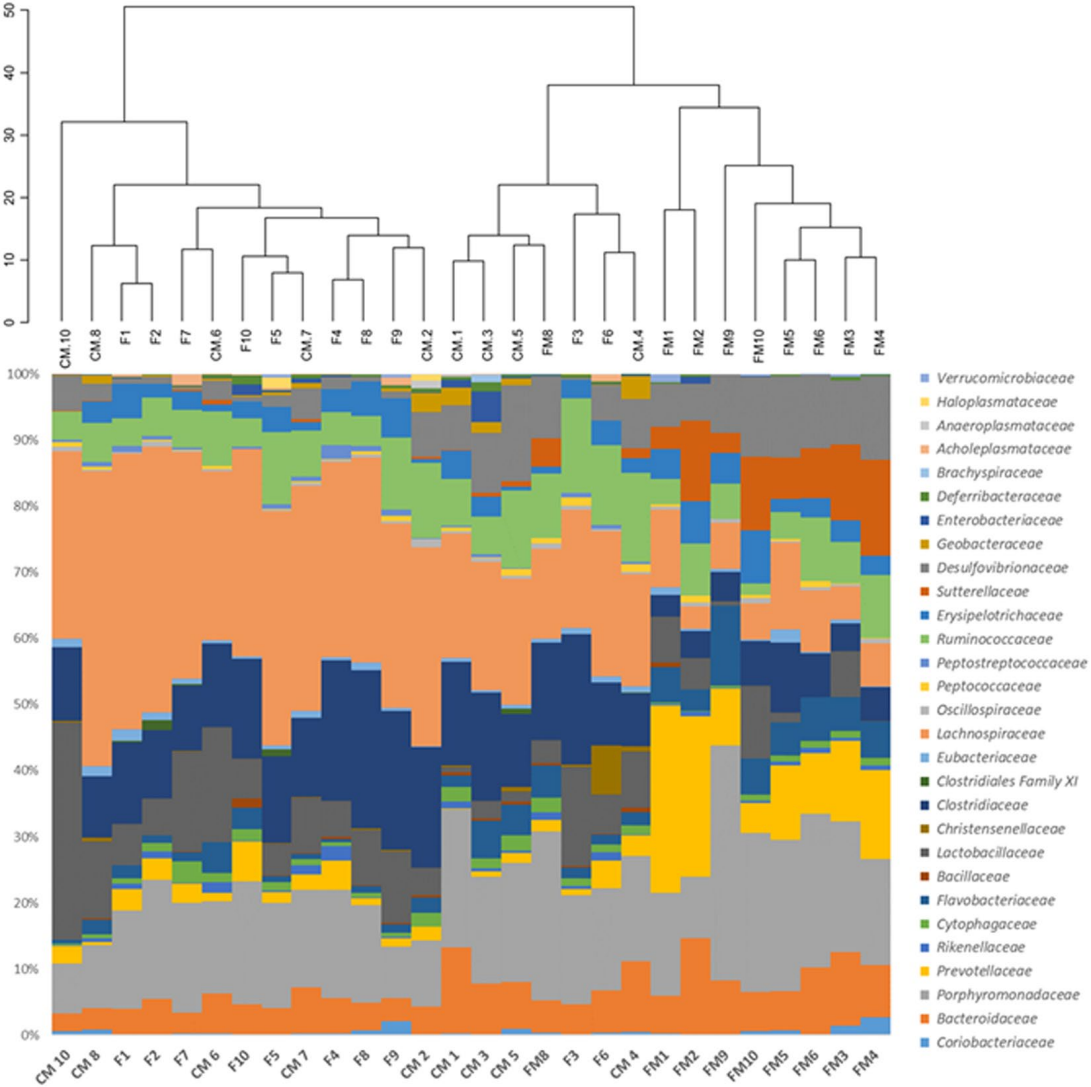
Differences in cecal microbiota composition at the family level for the rats belonging to the three cohorts in the genetic CRC animal model. F: feed cohort, CM: control meat cohort, FM: functional meat cohort.

The higher percentage of *Bacteroidetes* populations in the functional meat cohort was mainly due to a significant increase in the families *Bacteroidaceae*, *Porphyromonadaceae* and especially *Prevotellaceae* (Figs 6 and 7). These three families contain numerous genera involved in propionate production^56^, which may explain the higher propionate cecum concentration detected in the functional meat cohorts of both animal models. In some studies, the *Prevotellaceae* family has been associated with a higher health status, reduced CRC and better survival in CRC patients, due to its fermentative capabilities^54,55,57–61^. Also, the *Rikenellaceae* family (phylum *Bacteroidetes*) was reduced in the functional meat cohort, which is in accordance with other studies associating this family with CRC patients and potentially showing a pro-inflammatory effect in these situations^62,63^.

The marked reduction in *Firmicutes* phylum, observed in the functional meat cohort, was a consequence of the reduction of the *Lachnospiraceae, Ruminococcaceae* and *Clostridiaceae* families (Figs 6 and 7). Such reductions have been described in other studies when inulin is included in the diet^64,65^. Even though the *Lachnospiraceae* family suffered this reduction in the functional meat cohort, its *Blautia* genus showed a marked increase associated with the inulin diet (Tables 1 and 2). *Blautia*, associated with the fermentation of prebiotic fibers and with anti-inflammatory activity, has reduced populations in CRC patients^66–70^. In the case of the genetic CRC animal model, the *Lachnospiraceae* genera *Anaerostipes*, *Dorea* and *Lachnoclostridium* were increased in the functional meat cohort as well (Table 2). These three genera have been described as good propionate and butyrate producers^56,66,70^. Inside the *Ruminococcaceae* family, the main reduction at the genus level was observed in *Ruminococcus*, and more specifically in the *R. gnavus* species, which is a positive result since this species possesses virulence factors, is associated with bacteremia, and is higher in patients with IBD and other inflammatory conditions (such as allergies)^71–73^. The *Faecalibacterium* genus (a *Ruminococcaceae* member) was increased in the functional meat cohort of the genetic CRC animal model. *Faecalibacterium* is considered an anti-inflammatory species (via NF-κB inhibition) and a butyrate producer^74,75^. The populations of the *Clostridiaceae* family (and its *Clostridium* genus) were also reduced in the functional meat cohort, as has been described for other prebiotic diets^76,77^. The *Acetanaerobacterium* genus of this family was totally absent in the functional meat cohort. This genus has been associated with the production of enterolactones, a risk factor for adiposity, and metabolic syndrome^78^.

Despite the reduction of the *Firmicutes* phylum in the functional meat cohort, the families *Erysipelotrichaceae* and *Acidaminococcaceae* showed an increase. Members of the *Erysipelotrichaceae* family have been described as inulin fermenters^55,64,79^. However, here the increase in the *Acidaminococcaceae* family was associated mainly with an increase in the *Phascolarctobacterium* genus, which has been described as a good SCFAs producer, especially propionate via the succinate pathway^80^. Therefore, the increase observed in this genus correlates with the higher propionate cecum concentrations detected in this functional meat cohort (Fig. 5a,c). In addition, some studies associate this *Phascolarctobacterium* genus with anti-inflammatory effects, such as a reduction in the serum levels of C-reactive protein and lipopolysaccharide binding protein^81^.

Finally, the *Proteobacteria* phylum increased in the functional meat cohort (4.69%) with respect to feed (0.29%) and control meat (2.94%) cohorts. This increase was mainly associated with an increase in the *Parasutterella* genus (Tables 1 and 2). The genus *Parasutterella* is associated with the fermentation of prebiotic fibers and its populations are reduced in obese patients^82,83^. However another important genus of this phylum, *Desulfovibrio*, was reduced in the functional meat cohort (Tables 1 and 2). This genus has pro-inflammatory characteristics due to the production of H_2_S, the presence of lipopolysaccharide in its outer membrane, and the degradation of mucin. In addition, *Desulfovibrio* populations are increased in CRC patients^80,84–86^. In the genetic CRC animal model, there was a surprising increase in the *Bilophila* genus populations in the functional meat cohort (Table 2), which traditionally is associated with pro-inflammatory conditions, especially in mutant animals lacking IL-10^87–89^. However, its increase here was not associated with higher incidence of colon polyps.

In general, at the family level, the feed and control meat cohorts showed a similar pattern (high proportions of *Lachnospiraceae*, *Clostridiaceae* and *Porphyromonadaceae*), in contrast with functional meat cohort (high proportions of *Prevotellaceae*, *Bacteroidaceae* and *Porphyromonadaceae*) (Figs 6 and 7). Therefore, inulin supplementation was able to modulate cecum microbiota populations at phylum and family levels. These modulations caused by dietary inulin via functional meat foods were mainly associated with the reduction of pro-inflammatory cecum populations (such as *Ruminococcus, Clostridium, Acetanaerobacterium* or *Desulfovibrio*), and with an increase in the populations of taxons able to generate SCFAs from inulin fermentation (such as *Blautia, Faecalibacterium, Phascolarctobacterium* or *Parasutterella*).

Two other previous works in this animal model for studying colorectal polyps prevention have used a prebiotic preparation (GOS-Lu) and a functional meat product (sausages) including anthocyanidins from berry extract. The prebiotic preparation used (10% GOS-Lu in drinking water for 20 weeks) was able to reduce colon polyps up to 57.5% and polyps total area up to 50.4%, a similar reduction to the one achieved in this work using inulin functional meat products. In both cases an increase of microbial species known to generate SCFAs as propionate and butyrate (such as *Dorea, Blautia, Anaerostipes*) was observed in association with the prebiotic diet, as well as a reduction of some other populations with known pro-inflammatory properties (such as *Desulfovibrio*). In the case of the anthocyanidins-rich sausages, the antitumor preventive effect was observed between the functional sausages and feed control. This preventive effect was smaller than in the case of prebiotic functional foods, maybe due to the different mechanism of action, which in that case is not related to the production of antitumor SCFAs but to an antioxidant effect (measured as FRAP assay in that work)^30^.

**Figure 8:**
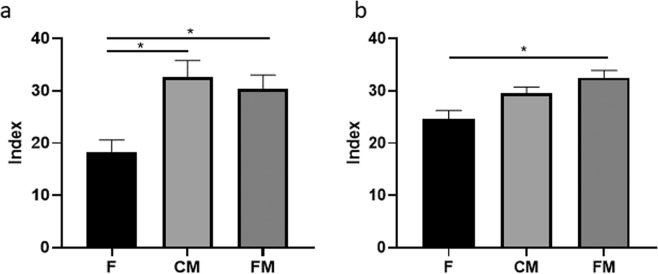
(**a)** Chao diversity index for the three rats cohorts in the wild type animal model. (**b**) Chao diversity index for the three rats cohorts in the genetic CRC animal model. F: feed cohort, CM: control meat cohort, FM: functional meat cohort.

In both animal models presented in this work, the presence of functional meat diets with inulin increased the diversity index (Fig. 8) in comparison with feed cohorts. Therefore, the two processed meat products involved in this study, after the redesign of their formulation towards functional prebiotic meat foods, could be useful in providing CRC prevention in human populations where they are traditionally consumed (Europe, America, Oceania), if their modulation in the human colon microbiota composition shows a similar pattern to the one observed in these two animal models, something that would require nutritional intervention studies in human populations.

## Materials and Methods

### Animals and experimental design

In the induced CRC animal model, 50 male Fischer 344 rats were maintained in the Animal Facilities at the University of Oviedo (authorized facility No. ES330440003591). The Ethics Committee of the Principality of Asturias (authorization code PROAE 16/2015) approved all rat experiments. All experiments were performed in accordance with relevant guidelines and regulations (Federation of European Laboratory Animal Science Associations, FELASA).

In this assay, the rats (five weeks old) were divided into five cohorts of 10 individuals each and fed ad libitum in individual cages. Cohort 1 was fed universal feed (2014 Teklad Global 14% Protein Rodent Maintenance Harlan diet feed, Harlan Laboratories, Barcelona, Spain). This feed contained 14.3% protein, 4% fat, 48% carbohydrates, 22.1% fiber, 4.7% ashes. The caloric content is 290 kcal/100 g (Table 5).

**Table 5:** Nutritional composition of the different diets used in this study.

	F	CC	FC	CH	FH
Protein	14.3%	30.74%	27.5%	24.6%	19.5%
Lipids	4%	11.9%	8.27%	9.8%	8.6%
Carbohydrates	48%	23.24%	17.34%	1.35%	1.15%
Fiber	22.1%	—	15.7%	—	10%
kcal/100 g	290	307	238	129.75	109.21

F: rat feed, CC: control *chorizo*, FC: functional *chorizo*, CH: control cooked ham, FH: functional cooked ham.

Cohort 2 was fed 10 g/day/rat of feed and 20 g/day/rat of control *chorizo* sausages (1 kg of porcine loin ribbon, 220 g porcine bacon, 20.5 g NaCl, 12.3 g dehydrated garlic, 22.1 g dehydrated pepper, 3 g dehydrated hot pepper). The loin ribbon composition was 30% protein, 8% lipids, 70% humidity, 0.1% ashes. Smoking was carried out using a low temperature wood friction method that avoids the presence of benzopyrenes in the final smoked food.

Cohort 3 was fed 10 g/day/rat of feed and 20 g/day/rat of control cooked ham (1 kg of porcine ham after removing the bone, injected with a brain solution containing NaCl, dextrose and dehydrated black pepper). Then the food matrix was subjected to massage/maturation and finally to molding and cooking at 65 °C. The final product (24.6% protein, 9.8% lipids, 2% NaCl, 0.4% dextrose) was refrigerated before proceeding to the slicing process.

Cohort 4 was fed 10 g/day/rat of feed and 20 g/day/rat of functional *chorizo* sausages of same formula but including 15.7 g inulin (Orafti HP, Beneo).

Cohort 5 was fed 10 g/day/rat of feed and 20 g/day/rat of functional cooked ham of same formula but with the addition of 10% inulin (Orafti HP, Beneo) during the brine injection process.

Nutritional compositions of the final produced products are described in Table 5. In the final formulas selected, taste and palatability were not changed by the presence of inulin in either food matrix. The parameters analyzed in these tests were color, general aspect, shape, smell, texture, hardiness (during chewing process), taste (sweet, salty, bitter, acid, unami), and flavor. Therefore, both functional meat products showed low fat indexes and high prebiotic properties.

In the genetic CRC animal model, 30 male KAD rats with F344/NSlc-Apc^1588/kyo^ genotype (SLC Inc., Japan) were maintained in the Animal Facilities at the University of Oviedo (authorized facility No. ES330440003591). 10 belonged to the feed cohort, 10 to the control *chorizo* cohort and 10 to the functional *chorizo* cohort. The Ethics Committee of the Principality of Asturias (authorization code PROAE 15/2017) approved all rat experiments.

Rats (five weeks old) were divided into five cohorts of 10 individuals each and fed ad libitum in individual cages. Cohort 1 was fed universal feed, as in the previous case. Cohort 2 was fed 10 g/day/rat of feed and 20 g/day/rat of control *chorizo* sausages, as in the previous case. And cohort 3 was fed 10 g/day/rat of feed and 20 g/day/rat of functional *chorizo* sausages, as in the previous case.

### Colorectal cancer induction and monitoring

As described previously^30^, the colorectal cancer experiment took place one week after the animals arrived at the facility, when the corresponding diets were started. After one week of eating the corresponding diet, CRC was induced in eight rats from each cohort. Only two control rats per diet were kept free of CRC induction as absolute control animals in each cohort. CRC induction was carried out in those eight rats of each cohort using azoxymethane (AOM, Sigma-Aldrich, Madrid, Spain) dissolved in sterile saline (0.9% NaCl) at a concentration of 2 mg/mL. This AOM solution was injected intraperitoneally at a final concentration of 10 mg per kg body weight. The AOM treatment was repeated seven days after the first injection (weeks 2 and 3). The absolute control animals received sterile saline in both injections^30,90^. This method for inducing colorectal cancer has been previously described^30^.

In weeks 4 and 15, rats received drinking water during a seven days treatment, containing 3% and 2% dextran sodium sulfate (DSS, 40.000 g/Mol, VWR), respectively. This ulcerative colitis step was repeated twice because it enhances the pro-carcinogenic effect caused by AOM administration. In the case of the genetic CRC animal model, the second DSS challenge (ulcerative colitis step) was not applied, in order to prevent further casualties in these animals due to intense rectal bleeding^30^.

Throughout the entire process, rats were continuously monitored for body weight and stool consistency/rectal bleeding.

### Physical measurements

Rats were weighed regularly during the 20 experimental weeks: at reception of the animals (week 1), after the first AOM administration (week 2), and at weeks 8, 13, 18 and 20.

### Blood and tissue samples

In both animal models, rats were anesthetized (isoflurane) before being sacrificed (bilateral pneumothorax) at week 20 after the first administration of AOM. This allowed for the extraction of 2 mL of blood from the beating heart during this deep anesthesia, which was then centrifuged at 3,000 rpm for 15 min, in order to collect and freeze the plasma. 30 µL of plasma from each animal were used for measuring triacylglycerides, using the Reflotron Plus (Roche, Madrid, Spain)^30,90^.

The small intestine was removed fresh and the hyperplastic Peyer’s patches (showing lymphoid hyperplasia, without atypia and not affecting adjacent tissues) were counted by naked eye, as these patches are larger than 2 mm in diameter. Their number in the experimental animals was calculated and compared to the small intestine Peyer’s patches of the two absolute control animals from each cohort (animals 9 and 10)^30,90^.

Cecums were weighed immediately after sacrifice using a precision scale and then frozen at −20 °C. Complete cecums were used for obtaining the weight data. Cecum content for SCFA and metagenomics analyses was removed after weight measurements^30,90^.

Finally, the colon was opened longitudinally and washed with PBS (phosphate buffer saline) before keeping it in 4% formaldehyde at 4 °C. Fixed colons were meticulously examined with a micrometer in order to count the number of polyps larger than 1 mm on the inner mucosa surface. For this, visual analysis was carried out, as all polyps larger than 1 mm diameter were counted. The largest detected polyps were 10 mm in diameter. The shape of the polyps was identified as pedunculated (a disc connected via a peduncle to the colon mucosa), plane irregular, plane circular and spherical^30,90^. Finally, the total polyps-affected area was calculated based on its shape^30^. Polyps were analyzed at the histological level by the Animal Histopathology Unit of the University of Oviedo. For this, colon samples were opened along the longitudinal axis and fixed for 24 h in 4% phosphate-buffered formaldehyde at room temperature before being embedded in paraffin blocks, in accordance with routine procedure. Specimens were sectioned in 5 µm thick sections and were stained using hematoxylin and eosin. Microscopic diagnosis was performed on microphotographs obtained by an Olympus BX-53 microscope and a DP73 digital camera connected to a computer with CellSens software.

In regard to the polyps’ classification, low degree dysplasia was defined in tissue areas showing hyperchromasia, pleomorphism, an increase in the nucleus/cytoplasm relationship and an increase in the proliferative activity affecting the two basal thirds of the epithelium. High degree dysplasia was detected in tissue areas showing the same alterations affecting the whole epithelium thickness. Adenocarcinoma was defined in tissue areas showing a malignant epithelial polyp with severe cytological atypia, large and pleomorphic nucleus with irregular nuclear membrane and one or several nucleoli (sometimes prominent). These adenocarcinomas can be intramucosal (when it is limited to the lamina propia), infiltrating (when it expands to the submucosa), low degree (with good differentiation) or high degree (with low differentiation). This method for classifying colorectal polyps has been previously described^30^.

### SCFAs analysis by CG-MS

Analysis and quantification of SCFAs in cecum content was carried out as described in the literature^90^.

### Genomic DNA extraction and 16S ribosomal RNA sequencing for metagenomics

Genomic DNA (gDNA) was extracted from 200 mg of frozen cecum feces using E.Z.N.A.^®^ DNA Stool Kit (Ref. D4015-02, VWR, Madrid, Spain) and provided 200 µL of genomic DNA. These gDNA samples were then quantified using a BioPhotometer^®^ (Eppendorf, Madrid, Spain) and their concentrations diluted to 6 ng/µL. The diluted samples were used for performing polymerase chain reaction (PCR) amplification, following the protocol of the Ion 16^TM^ Metagenomics kit (Thermo Fischer Scientific, Madrid, Spain)^30^.

PCR amplification products were utilized to create a library using the Ion Plus Fragment Library kit for AB Library Builder^TM^ System (Cat. No. 4477597, Thermo Fischer Scientific, Madrid, Spain), with sample indexing using the Ion Xpress^TM^ Barcode Adapters 1-96 kit (Cat. No. 4474517, Thermo Fischer Scientific, Madrid, Spain). Template preparation was performed using the ION OneTouch^TM^ 2 System and the ION PGM^TM^ Hi-Q^TM^ OT2 kit (Cat. No. A27739, Thermo Fischer Scientific, Madrid, Spain). Metagenomics sequencing was performed using ION PGM^TM^ Hi-Q^TM^ Sequencing kit (Cat. No. A25592, Thermo Fischer Scientific, Madrid, Spain) on the ION PGM^TM^ System. The chips used were the ION 314^TM^ v2, 316^TM^ v2 or 318^TM^ v2 Chips (Cat. No. 4482261, 4483188, 4484355, Thermo Fischer Scientific, Madrid, Spain) with various barcoded samples per chip^90^. This method for analyzing metagenomics data has been previously described^30^.

### phylogenetic analysis

The consensus spreadsheet for each metagenomics sequencing was downloaded from ION Reporter software (version 5.6, Life Technologies Holdings Pte Ltd, Singapore). This spreadsheet includes the percentages for each taxonomic level and was used for comparing frequencies between individual rats and cohorts. Analyses were carried out using QIIME-2 software^30,90^.

### Statistical methods

The normality of the different variables was tested using Shapiro–Wilk’s test. In light of these results, the data were then expressed as the mean value ± standard error of mean (SEM) and parametric methods were used for statistical analyses. The equality of variances was tested using Levene’s test. In the case of the chemically induced CRC animal model, the statistical analyses demonstrated that there was equality of means for the parameters cecum weight, number of hyperplastic Peyer’s patches, number of polyps, total polyps area, SCFAs concentrations and metagenomics analyses (phyla, families, genera and species composition). The differences among cohorts were tested by a one-way ANOVA (analysis of variance)^90^, which showed no statistical differences between chorizo and cooked ham cohorts of each type (control or functional). Therefore, in order to evaluate results with more *n* per cohort, all further analyses were carried out taking into consideration just three cohorts (instead of five): feed, control meat (control *chorizo* and control cooked ham) and functional meat (functional *chorizo* and functional cooked ham), each one of 10, 20 and 20 animals respectively.

The graphic representation of all the data was carried out using GraphPad Prism software, (version 7, GraphPad Software, San Diego, CA, USA). In each case, a *p* value < 0.05 was considered statistically significant (*p < 0.05)^30,90^.
